# Hierarchical Stereo Matching in Two-Scale Space for Cyber-Physical System

**DOI:** 10.3390/s17071680

**Published:** 2017-07-21

**Authors:** Eunah Choi, Sangyoon Lee, Hyunki Hong

**Affiliations:** 1 Department of Imaging Science and Arts, GSAIM, Chung-Ang University, 221 Huksuk-dong, Dongjak-ku, Seoul 156-756, Korea; eunazzy@naver.com; 2 School of Integrative Engineering, Chung-Ang University, 221 Huksuk-dong, Dongjak-ku, Seoul 156-756, Korea; leesy88@cau.ac.kr

**Keywords:** stereo matching, scale space image, disparity map, difference of Gaussian, Canny edge detector, cost aggregation

## Abstract

Dense disparity map estimation from a high-resolution stereo image is a very difficult problem in terms of both matching accuracy and computation efficiency. Thus, an exhaustive disparity search at full resolution is required. In general, examining more pixels in the stereo view results in more ambiguous correspondences. When a high-resolution image is down-sampled, the high-frequency components of the fine-scaled image are at risk of disappearing in the coarse-resolution image. Furthermore, if erroneous disparity estimates caused by missing high-frequency components are propagated across scale space, ultimately, false disparity estimates are obtained. To solve these problems, we introduce an efficient hierarchical stereo matching method in two-scale space. This method applies disparity estimation to the reduced-resolution image, and the disparity result is then up-sampled to the original resolution. The disparity estimation values of the high-frequency (or edge component) regions of the full-resolution image are combined with the up-sampled disparity results. In this study, we extracted the high-frequency areas from the scale-space representation by using difference of Gaussian (DoG) or found edge components, using a Canny operator. Then, edge-aware disparity propagation was used to refine the disparity map. The experimental results show that the proposed algorithm outperforms previous methods.

## 1. Introduction

A cyber-physical system (CPS) consists of various physical and software components, such as smart grids, autonomous automobile systems, process control systems, robotics systems, and automatic pilot avionics. CPSs monitor the physical processes, make decentralized decisions and trigger actions, communicating and cooperating with each other and with humans in real time [1,2]. 

Vision systems have been widely used in applications related to improving the safety of workers in the industrial environment and for robot guidance. Autonomous automobile and mobile robotics systems require 3D depth information to interact with the real world or human beings. Stereo vision is used to obtain an accurate and detailed 3D representation of a real scene in a manner similar to the human vision system. A stereo camera can help automobile and mobile robotics systems navigate a real environment better by using depth to segment the objects of interest and background elements. The depth information is combined with monocular image features to better recognize and match features between consecutive frames. In addition, a hybrid stereo matching system that combines active and passive stereo vision was proposed to handle untextured regions [3]. 

Stereo matching algorithms are broadly classified into local and global matching methods [4]. The former sums pixelwise matching costs, whereas the latter supports piecewise smooth disparity selection at every pixel simultaneously [4,5,6,7,8,9,10,11,12,13,14,15,16,17]. Global methods define a global energy function that includes a data term and a smoothness term. In order to find the minimum of the global energy function, various global optimizers such as dynamic programming (DP) [5,6], belief propagation [7], and graph cuts [8] are used. Most global stereo methods are computationally expensive and involve many parameters. Local algorithms select the disparity hypothesis with the minimal matching cost at the pixel and are therefore efficient and easy to implement. In addition, stereo matching methods consist of four individual components: matching cost computation, cost aggregation, disparity computation, and disparity refinement [4]. In stereo vision, the recovery of an accurate disparity map remains a challenge because of occlusion problems, illumination effects and imaging sensor noise.

The goal of stereo vision is to determine a precise disparity that indicates the difference in the corresponding pixel position between stereo views. A higher resolution of the examined image results in more correct depth information being obtained. However, as the image resolution increases, the computation load required to establish correspondence points increases. Furthermore, the more pixels in the stereo view are examined, the greater the number of matching candidate pixels, and the matching ambiguities are considerably increased. A few stereo matching methods have recently been proposed to handle high-resolution images in near-real time [9,10,11]. This paper presents a hierarchical method to obtain a reliable disparity map of high-resolution stereo images in near-real time. 

To reduce the search space of a block matching algorithm and the redundant contents in image compression, multi-scale image representation has been used actively. In stereo vision, multi-scale representation, called the coarse-to-fine approach, has been used mainly to achieve computational efficiency [12,13,14,15]. First, a full-resolution image is scaled down and a disparity range search of the reduced-resolution image is performed efficiently. Then, the disparity values obtained are scaled up to the image’s original resolution. By using coarse-to-fine approaches, computation performance can be improved significantly compared to that of an exhaustive disparity search at full resolution [12]. However, while image smoothing and sub-sampling are performed in discrete scale-space conversion, the high-frequency components of fine-scaled images are at risk of disappearing in a coarse-resolution image. Therefore, when disparity estimates are propagated across scale space in the coarse-to-fine refinement procedure, the disparity estimation results become erroneous. 

Geiger built a priori information about the disparity value by forming a triangulation on a set of support points that can be robustly matched [9]. This allows efficient utilization of the disparity search space and a reduction of the matching ambiguities of the remaining points. However, the priors based on the support points are likely to fail to reconstruct poorly-textured and slanted surfaces. Sinha employed local slanted plane sweeps for disparity hypotheses in semi-global matching [10]. The local plane hypotheses are derived from initial sparse feature correspondences, and the local plane sweeps are then performed around each slanted plane to produce a globally optimal disparity value. In general, disparity plane estimation is a time-consuming procedure and additional consideration of large and untextured areas is needed.

Zhao described a progressive multi-resolution pipeline that includes background modeling and dense matching with adaptive windows [11]. To achieve a high-resolution disparity map, the stereo search area is limited to the span range that is centered at the suggestion from the disparity estimation values in the lower resolution. This coarse-to-fine approach can improve computation performance significantly. However, the method is at risk of falling into a local minimum in the case of a real-world scene composed of complex objects and backgrounds. In other words, when a false matching result is obtained from the coarse-resolution image, the matching accuracy deteriorates considerably because of false disparity suggestion over scale space. 

**Figure 1:**
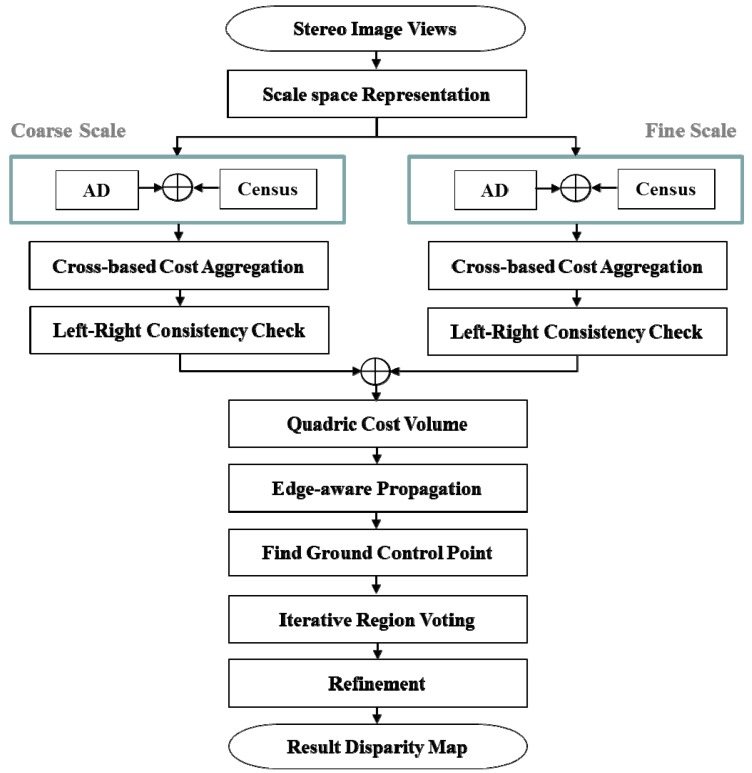
Proposed block diagram.

In two-scale space representation (both coarse- and fine-resolution stereo images), the method proposed computes the initial matching costs by applying absolute difference (AD)-census and then aggregates the matching costs in cross-based support regions [16,17]. By using difference of Gaussian (DoG), the method proposed obtains high-frequency regions from the full-resolution image. DoG is the difference of two Gaussian averaging masks with substantially different standard deviation parameters [18]. In addition, the edge components in the original resolution image are detected by a Canny operator. In the original resolution image, we compute the disparity value from only either the high-frequency regions or the edge components. In order to discriminate the inliers and the outliers (occluded pixels and false-matching pixels), a left-right consistency (LRC) check is employed in the two-scale stereo image. The disparity results in the coarse scale are up-sampled into the full-resolution image, and the up-sampled disparity results are combined with the disparity estimation results in the fine-resolution image. Here, the DoG intensity ratio with respect to the maximum DoG value is used. In the case of edge components, the rectangular area around the obtained edge can be used for disparity estimation. Edge-aware disparity propagation (EDP), where the disparities are propagated in the cost domain, is used as geodesic filtering based on edge cost information [19]. Finally, iterative voting with reliable ground points and refinement are employed. We demonstrate the effectiveness of our approach in terms of both accuracy and computational speed using large-resolution Middlebury benchmark images. Figure 1 shows the block diagram of the proposed algorithm.

## 2. Proposed Method 

A hierarchical stereo matching method provides an efficient coarse-to-fine mechanism to estimate 3D depth information. The high-frequency components of a fine-resolution image are at risk of disappearing in the down-sampling procedure. In addition, when erroneous disparity estimates are propagated across scale space, false disparity estimates are obtained. Since the maximum disparity level (the search space) increases in a high-resolution image, the likelihood of false disparity estimation results also increases. In order to solve these problems, we efficiently combine the disparity estimation results in the coarse-resolution image with the disparity results in high-frequency regions of the fine-resolution image. 

### 2.1. Initial Matching Cost Computing

Given an anchor pixel **p** = (*x*, *y*) in the left image (reference view) *I^Left^* and its candidate correspondence **pd** = (*x* − *d*, *y*) in the right image (target view) *I^Right^*, two individual cost volumes *C_AD_*(**p**, *d*) and *C_census_*(**p**, *d*) are computed. The two cost volumes are combined to obtain an initial matching cost *C_initial_*(**p**, *d*) along the disparity range *d* [16]. *C_AD_*(**p**, *d*) is defined as the average intensity difference of **p** and **pd** in RGB channels in Equation (1). On the assumption that input stereo images are rectified, the *y* coordinate component is usually omitted.

(1) $$C_{AD}(p,d)=\frac{1}{3}\sum_{i=R,G,B}\left|I_{i}^{Left}(p)-I_{i}^{Right}(pd)\right|$$

(2) $$C(p)\;=\;\underset{p_{n}\in N(p)}{⊗}\xi (p,\;p_{n}),\;\xi (p,\;p_{n})=\{\begin{array}{c} 1,\;\mathrm{if}\;p<p_{n}\\ 0,\;\mathrm{otherwise} \end{array}$$

(3) $$C_{initial}(p,d)\;=\;\rho (Ccensus(p,d),\lambda census)\;+\;\rho (C_{AD}(p,d),\lambda_{AD}),\;\rho (c,\lambda )\;=\;1-\exp(-\frac{c}{\lambda })$$

**Figure 2:**
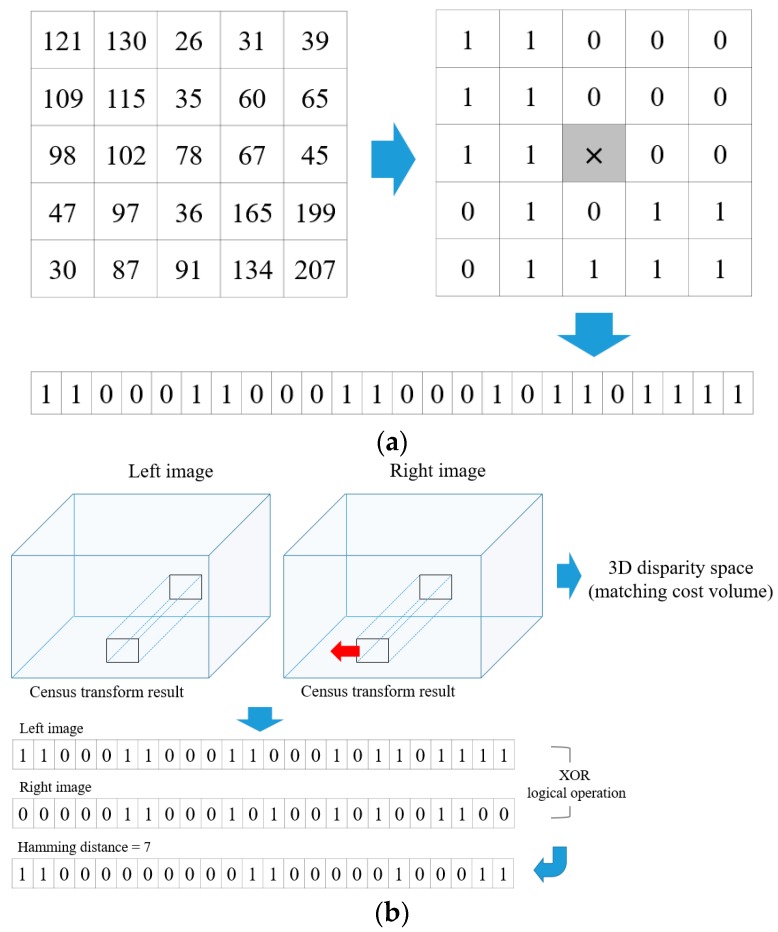
(**a**) Example of census transform; (**b**) Its hamming distance result.

By comparing the brightness value of the neighborhood pixel with that of an anchor pixel, census transform converts the relative orderings of the pixel intensities to binary strings. The bit string *C*(**p**) is computed as a value compared by the operator ⊗, subjected to the brightness value of anchor pixel **p**, and the brightness value of neighborhood pixel *N*(**p**) in a census window of a fixed size and shape. *C_census_*(**p**, *d*) is defined as the Hamming distance of the two bit strings **p** in the left image and its correspondence **pd** in the right image. Figure 2 shows an example of census transform and its Hamming distance result, which is used to build the 3D disparity space. In this study, a 9 × 7 rectangular window was employed for census transform. The estimation results yielded by the AD and the census transform methods are combined in an exponential function, as shown in Equation (3). This function maps different cost measures to the range [0, 1] and controls easily the influence of the outliers with the *λ* parameter. Here, *λ_AD_* and *λ_census_* are set to 10 and 30, respectively.

Because the AD measure examines only the pixel intensity, it is affected significantly by illumination changes. The census transform encodes local image structures with relative orderings of the pixel intensities rather than the intensity value itself, in order to tolerate outliers that are caused by radiometric changes and image noise. Instead of using color difference between image views, we calculate the absolute difference of the Laplacian transformed images to alleviate the unwanted effects of the camera’s parallax. The initial matching costs of the AD-census method are aggregated in cross-based support regions [17]. For each pixel, an upright cross local support skeleton region is adaptively constructed by considering the color similarity, as well as the connectivity constraints.

More specifically, an upright (the vertical and horizontal) cross with four arms is constructed for an anchor pixel **p**. The endpoints **p***_e_* of the horizontal and vertical directions with respect to the pixel **p** are determined by examining the color distance *D_c_*(**p**, **p***_e_*) and the spatial distance *D_s_*(**p**, **p***_e_*), as shown in Equation (4). By computing the color difference between **p***_e_* and its predecessor **p***_e_* + 1 along the scanning direction (left, right, up, and bottom) of each arm, the arm does not run across the edges in the image. Then, the support region for pixel **p** is modeled by merging the horizontal arms of all the pixels lying on **p**’s vertical arms. The matching costs of the AD-census measure are summed horizontally and stored as the intermediate results. Then, the intermediate results are aggregated vertically to obtain the final costs. Both passes can be efficiently computed with 1D integral images [17].

(4) $$D_{c}(p_{e},p)\;<\tau_{1}\;\mathrm{and}\;D_{c}(p_{e},p_{e+1})\;<\tau_{1},\;D_{s}(p_{e},p)\;<L_{1},\;D_{c}(p_{e},p)\;<\tau_{2}\;\mathrm{if}\;L_{2}<D_{s}(p_{e},p)\;<L_{1}$$

This paper presents a method to compute the initial matching costs using adaptive windows. Most methods obtain initial matching costs with a rectangular shaped window, regardless of the local color (intensity) distribution. Disparity estimation results yielded by fixed-size windows are influenced frequently by irrelevant pixels within the window region under consideration. In order to reduce the erroneous effects caused by irrelevant pixels, Kanade introduced adaptively shaped windows [20]. Adaptive window approaches focus on varying the size, shape, and position of the window. The adaptive-weight method to reduce the unwanted effects caused by irrelevant pixels within a local window gives a varying support weight for each pixel in the window. Different support weights are assigned to pixels in the window by evaluating the photometric and geometric relationship with the pixel under consideration [21]. However, many problems, including textureless regions, repeated similar patterns, and occlusions, remain unsolved. 

The method proposed employs the cross-based support region as a matching window to compute an initial matching cost. In general, it takes a considerable amount of time to build windows of variable size and shape that are adaptive to local intensity distribution. Because cross-based support regions have been widely used in initial cost aggregation, we can build adaptive windows without additional computation. First, the cross-based support regions of an anchor pixel in the reference image and in the target image are built along disparity levels. In order to compare the pixel correlation of stereo views in the same-sized local areas accurately, we obtain an intersection region between support regions in the reference and target images.

**Figure 3:**
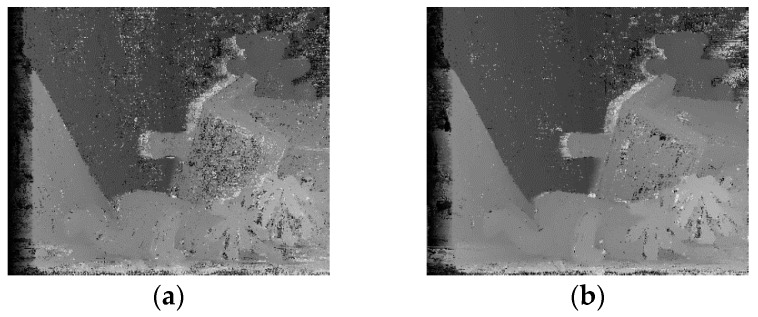
Initial matching cost results of (**a**) absolute difference (AD)-census and (**b**) sum of absolute difference (SAD)-census.

By using sum of absolute difference (SAD) and census transform methods in the intersection area, we compute the initial matching cost of the anchor pixel. Figure 3 shows the disparity value maps produced by the AD-census and the SAD-census method, respectively. Here, the disparity value with the minimum initial matching cost is obtained using the winner-takes-all (WTA) method. The disparity map produced by the SAD-census method is more precise than that produced by the AD-census method. The matching costs based on individual pixels are aggregated using a 3 × 3 window. The average costs within a squared window anchored at different locations are replaced with the cost of a given pixel [4].

### 2.2. Matching Cost in Scale Space

Since the maximum disparity level (the search space) increases in a high-resolution image, false disparity results may be obtained from poorly textured regions and regions with a repeated pattern. To overcome this problem, a stereo matching process is applied only to the pixel in the high-frequency component of the high-resolution stereo image. In general, high-frequency areas correspond to detailed parts of the scene and the edge regions of the objects, which are suitable for local stereo matching. The method proposed extracts the high-frequency areas from the scale-space representation by DoG or finds edge components by applying a Canny operator. DoG is obtained by subtracting one blurred version from another less blurred version of the original. In general, the blurred images are obtained by convolving the original grayscale images with Gaussian kernels having differing standard deviations. In this study, DoG was used to identify the edge regions and other detailed parts present in the full-resolution image. 

First, the proposed hierarchical method computes the initial matching cost of every pixel in the coarse-resolution image. In the full-resolution image, we compute the initial matching cost of only the pixel in the high-frequency regions or edge components. Then, the matching costs of the two-scale images are combined in fine scale. The disparity hypothesis having the minimum matching cost is determined as the final disparity value by using the WTA method. An LRC check is used to examine outliers that are caused by environmental lighting changes, background effects, and occlusions. This procedure is performed by taking the computed disparity value in one image and re-projecting it onto the other image. 

In scale-space representation, the image size increases exponentially as the scale level decreases. This means that the computational load of stereo matching in finer resolution increases exponentially. In other words, the maximum disparity level increases in a high-resolution image. An erroneous disparity estimation value may be propagated in a hierarchical stereo matching approach. To overcome these problems, we propose a hierarchical method that combines the disparity estimation results that are obtained in the coarse-resolution image with those for high-frequency components in the full-resolution image. 

In Equation (5), the disparity results *D_coarse_*(**p′**) at pixel **p′** in the coarse-resolution image are up-sampled to those in the full-resolution image. This means that the disparity value in the coarse scale is doubled. In the resolution pyramid, 2 × 2 pixels **p**s in the fine-scale image are merged to a single new pixel **p′** in the coarse-resolution image. If **p** is in the high-frequency regions, the disparity value *D_fine_*(**p**) is chosen as the final disparity result in the fine scale. Otherwise, the disparity value in the coarse scale is up-sampled:

(5) $$D(p)=\{\begin{array}{cc} D_{fine}(p), & \mathrm{if}\;p\;\mathrm{is}\;\mathrm{high}\;-\mathrm{frequency}\mathrm{component}\\ D_{coarse}(p^{\prime })\times 2, & \mathrm{otherwise} \end{array}$$

(6) $$D(p)=w\cdot D_{fine}+(1-w)D_{coarse}\;,\;w=\frac{\mathrm{DoG}\;\mathrm{value}\;\mathrm{at}\;p}{\mathrm{Maximum}\;\mathrm{of}\;\mathrm{DoG}\;\mathrm{value}}$$

**Figure 4:**
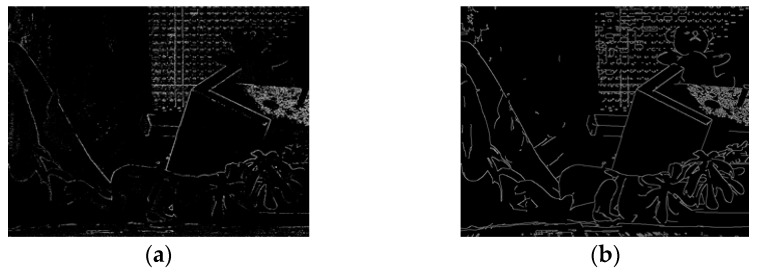
(**a**) High-frequency regions produced by difference of Gaussian (DoG) and (**b**) edge components produced by Canny operator.

When DoG is employed in scale-space representation, we compute the relative weight value *w*, as in Equation (6), where *w* is computed by dividing the DoG value at pixel **p** with the maximum DoG value in the image. To combine the disparity results in scale-space representation, we can apply a Canny operator to the fine-resolution image. The edge components found by the Canny operator are used as the high-frequency region in DoG, as in Equation (5). Figure 4 shows the high-frequency regions produced by DoG and the edge components produced by the Canny operator, respectively.

### 2.3. Disparity Refinement

An LRC check is performed to determine reliable pixels and their disparity candidates. In order to improve the stereo matching performance, we apply a 1D disparity propagation technique based on reliable pixels. Specifically, the obtained disparity values are propagated along the scanline direction to produce accurate and dense disparity results. Then, the EDP method is used to preserve the edges in the disparity propagation within near-real time [19].

**Figure 5:**
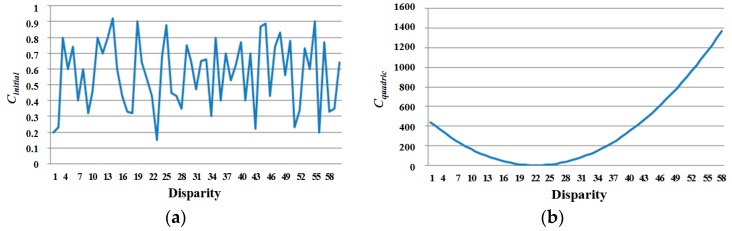
(**a**) Initial matching cost; (**b**) new quadric cost.

By using the modified penalty function for disparity varying from the per-pixel candidate subset, we compute the quadric cost of the stable pixel again. EDP makes the initial matching cost more distinguishable for determining a reliable disparity value, as shown in Figure 5. Given a disparity value *D*(**p**) and a per-pixel assignment label (stable or unstable), a new quadric cost is computed for each pixel **p** at each disparity level *d*:

(7) $$C_{quadric}(p,d)=\{\begin{array}{cc} |d-D(p){|}^{2} & \mathrm{if}\;p\;\mathrm{is}\;\mathrm{stable}\\ 0 & \mathrm{otherwise} \end{array}$$

**Figure 6:**
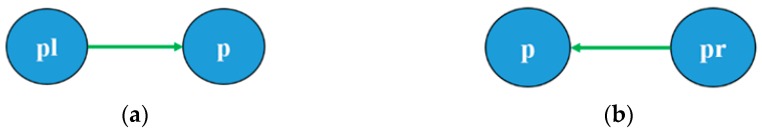
(**a**) First pass, from left to right; and (**b**) second pass, from right to left [19].

Then, we propagate the disparity values from the stable pixels to the unstable pixels, considering the spatial distance and edge similarity. For the first scanning pass from left to right, the cost value of a pixel **p**, *C_EDP_*(**p**, *d*), is sequentially updated:

(8) $$C_{EDP}(p,d)\;=\;C_{quadric}(p,d)+\alpha (p,pl)\cdot C_{quadric}(pl,d)$$

(9) $$\alpha (p,pl)\;=\;\exp(-\frac{1}{\sigma_{s}}-\frac{\Delta (p,pl)}{\sigma_{r}})$$

where **pl** is a left neighboring pixel **p**. *α*(**p**,**pl**) consists of the spatial proximity and the color similarity between **p** and **pl**. An edge cost *∆*(**p**,**pl**) is the maximum of the absolute differences computed separately in RGB channels between pixel **p** and **pl**. *σ_s_* and *σ_r_* are two parameters that can be used to balance the spatial and color components, respectively. In this study, *σ_s_* and *σ_r_* were experimentally set to 42.5 and 22.5. In the second scan from right to left, each pixel collects cost values on its right side, as in Equation (8). This 1D filter is then applied vertically on the result that is produced by horizontal passes. The 1D horizontal geodesic filter is applied via a two-pass aggregation, as shown in Figure 6.

To solve the occlusion problem, we proceed with iterative region voting, proper interpolation, and median filtering in that order. The pixels that pass the LRC check are determined as ground control points, representing reliable pixels. Otherwise, the pixels are determined as outliers. The outliers detected by the LRC check are filled with reliable neighbor points (ground control points) by using iterative region voting. This means that the disparity of the outlier is replaced with the disparity of the highest bin value (most votes) in the support region. The outliers discovered are classified into occlusion pixels and miss-matched pixels. Two types of outlier are handled by two interpolation strategies separately. For an outlier, we find the nearest reliable pixels in the neighborhood. We examine the disparity distribution in both the left and right directions around the outlier pixel discovered. If the number of the outliers discovered in the horizontal direction (in a row) is the same as the disparity value difference between the background and foreground object, we determine the pixels as outliers by occlusion. When the outlier is determined as an occlusion point, the pixel is likely to belong to the background. Therefore, the neighboring pixel with the lowest disparity value is selected for interpolation. Finally, a 3 × 3 sized median filter is employed to handle noise and miss-matched pixels.

## 3. Experimental Results

The following computing platform was used in the experiment: a PC (HP, Chongqing, China) with an Intel Core i7 3.07 GHz CPU and an NVidia GTX780 780Ti phantom D5 graphics card. The proposed system was tested using the Middlebury 2001, 2003, and 2006 benchmark datasets [22] and implemented on a GPGPU with CUDA that can be used to handle the heavy computational loads for stereo matching over the scale space. 

**Figure 7:**
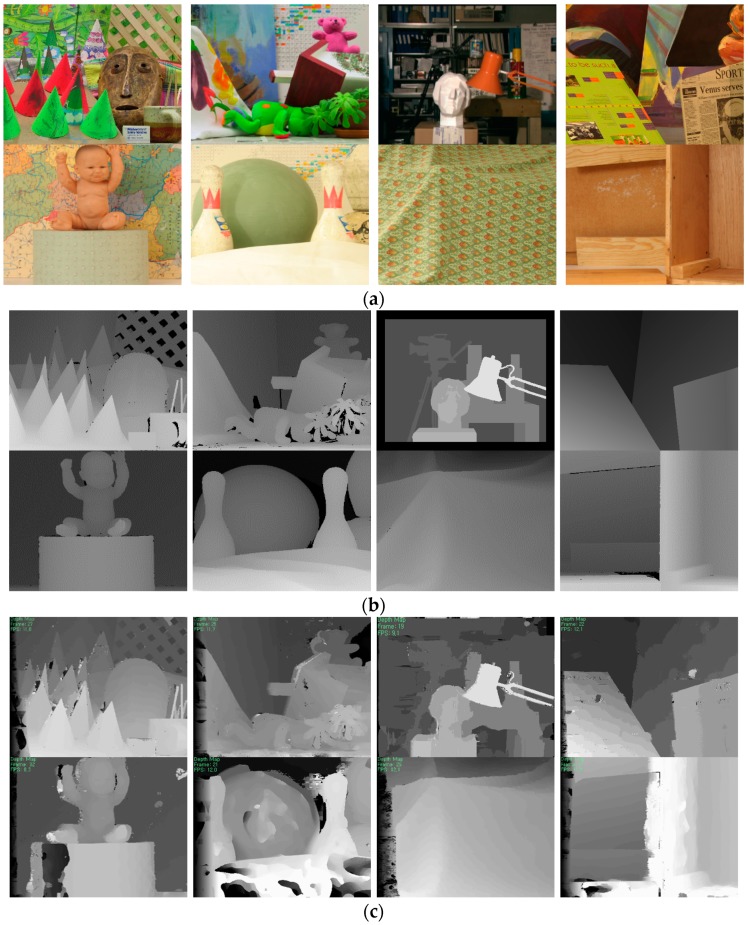

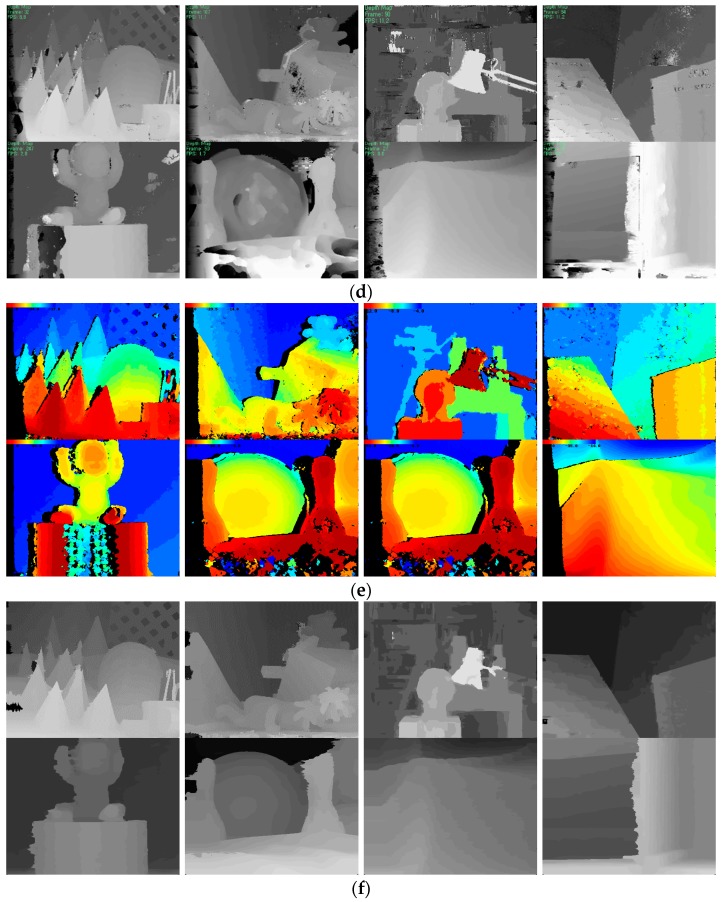
(**a**) Reference images (Middlebury) and (**b**) ground truth disparity maps; Disparity maps by (**c**) dual-cross-bilateral (DCB) grid [23]; (**d**) adaptive weight [21]; (**e**) MGM [24]; and (**f**) proposed method.

The method proposed was compared with previous algorithms qualitatively [21,23,24]. Figure 7a,b show Cones (900 × 750), Teddy (900 × 750), Tsukuba (384 × 288), Venus (434 × 383), Baby1 (1240 × 1110), Bowling (11250 × 1110), Cloth1 (1252 × 1110), and Wood1 (1372 × 1110) stereo images, and their ground truth depth maps, respectively. In the more global matching (MGM) method [24], a refinement procedure is not included and the disparity results are expressed in pseudo color. However, we can see the matching performance of the MGM method roughly. Figure 7 shows that the method proposed for combining disparity estimation results in two-scale space can improve disparity estimation performance. Here, the edge components produced by the Canny detector in the full-resolution image are used for disparity estimation. In the results (5th and 6th columns) of the method proposed, the spatial and color control parameters in EDP were set to 20 and 10 and 42.5 and 22.5, respectively. A previous method assigned different support weights to pixels in the window by evaluating the photometric and geometric relationship with the pixel under consideration [21]. Figure 7d shows that many erroneous disparity estimation results from textureless regions and repeated similar patterns are obtained. 

Table 1 shows a comparison of the quantitative evaluation results produced by stereo matching algorithms with the near-real time computation performance. Three Middlebury stereo images (Baby3 (1312 × 1110), Art (1300 × 1110), and Lamp2 (1300 × 1110)) were added. High-frequency regions (or edge component regions) frequently coincide with depth discontinuous regions. However, a discussion of the occluded regions is beyond the scope this paper, and occlusion problems will be considered further in future studies. Here, the threshold values for the mean squared errors of the ground truth map and the resulting disparity map were set to 1 and 2, respectively. The matching errors of previous methods were reported in the Middlebury benchmark. Table 1 shows that the method proposed provides better results than any of the other methods. In Table 1, the results of the error rate (%) achieved by the Canny detector are better than those obtained by DoG. Here, the best numerical results are marked in bold. When highly textured objects exist in the scene (Cones and Baby3), the best performance (the smallest errors) is obtained. In contrast, the worst performance is obtained for the Lamp2 image pair, since it is poorly textured.

**Figure 8:**
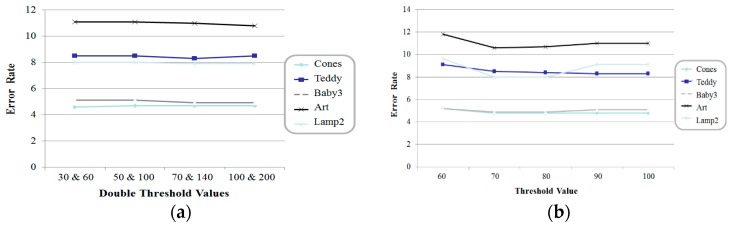
Comparison of error rates with respect to the threshold values of (**a**) Canny detector and (**b**) DoG method.

Figure 8 shows a comparison of the error rates with respect to the threshold values of the Canny detector and DoG method. When the double threshold values of the Canny detector were set to 100 and 200, the best performances were obtained in this experiment. In the case of DoG, a threshold value of 100% means that all the disparity values in high-frequency components are used to combine the disparity values of the two-scaled images. The method proposed obtains a more precise disparity map of a high-resolution image by considering the high-frequency components in two-scale space.

**Figure 9:**
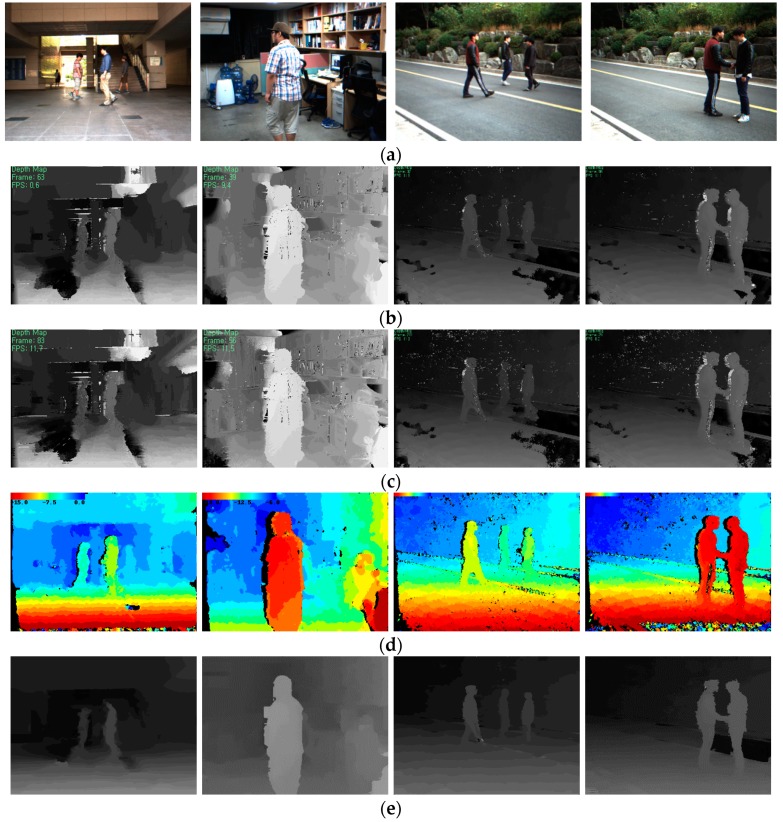
(**a**) Outdoor and indoor scene images; Disparity maps produced by (**b**) DCB grid [23]; (**c**) adaptive weight [21]; (**d**) MGM [24]; and (**e**) proposed method.

**Figure 10:**
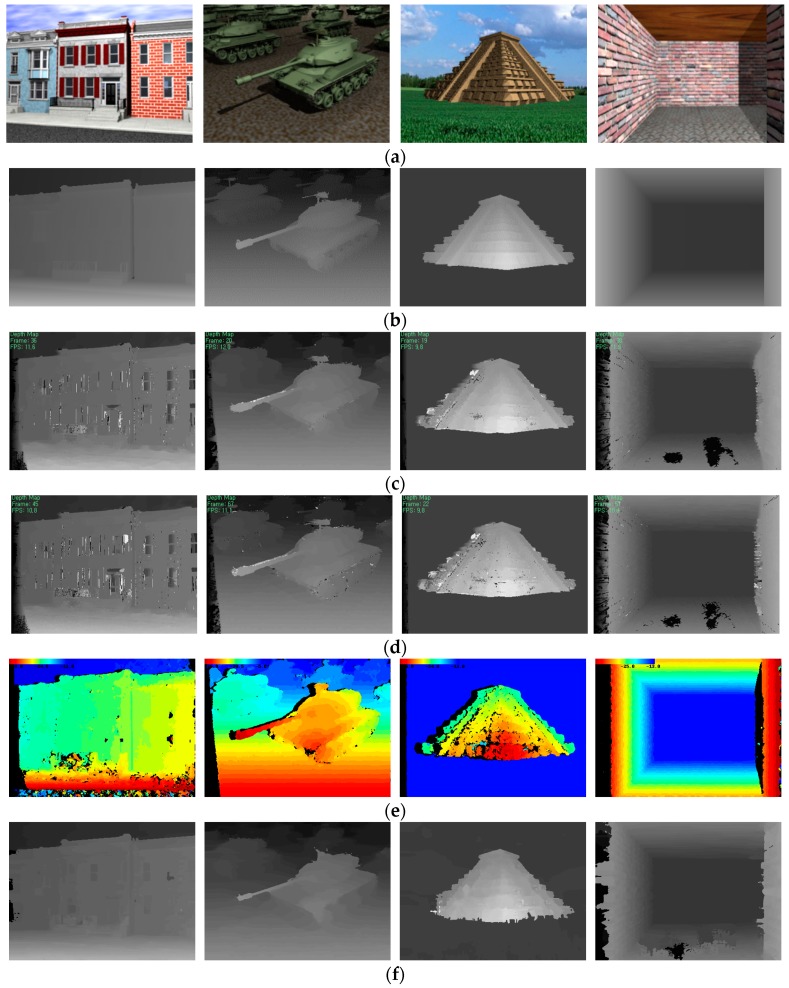
(**a**) Computer generated images and (**b**) ground truth disparity maps [23]; Disparity maps by (**c**) DCB grid [23]; (**d**) adaptive weight [21]; (**e**) MGM [24]; and (**f**) proposed method.

The experiment was performed using an outdoor and indoor scene image sequence, which is a real-world stereo video clip captured in an uncontrolled environment, as shown in Figure 9. Figure 10 shows the comparison results of the stereo methods for computer-generated images and their ground truth disparity maps [23].

The entire algorithm was implemented on a general-purpose computing on graphics processing units (GPGPU) using the Compute unified device architecture (CUDA) language, and could achieve a rate of over 5.0 frames per second for stereo images of 1300 × 1100 pixels. If our program is optimized in the near future, the computational time performance will be considerably improved. The method proposed is designed to obtain a reliable disparity map of high-resolution stereo images in near-real time. Therefore, two previous methods [9,10] for the same purpose are described and compared in terms of computation performance. The running time reported in [9] for a 900 × 750 pixel image was 669 msec, and the averaged running time in [10] for 1.4 megapixels (MP) to 2.7 MP was about 3.8 sec. In conclusion, the method proposed achieves a better performance in terms of computational speed than two algorithms.

Let the width and the height of an input image and the maximum disparity level be *W*, *H*, and *D*, respectively. In this case, stereo matching operations of Θ(*W* × *H* × *D*) are performed. Therefore, dense stereo matching in high-resolution stereo images requires a high computational cost. In contrast, the method proposed performs the Θ(*W* × *H* × *D*/8) operation for the reduced-resolution image and Θ(*W W* × *H* × *DF* × *D*) for the full-resolution image. Here, *HF* is the relative averaged ratio of the total area of the high-resolution image to the area of high-frequency regions. In our experiment, *HF* was set to 3.4%, which is the averaged area ratio of the Middlebury stereo images. For example, the number of stereo matching operations for the Lamp2 image with a 1300 × 1100 resolution with a 260 disparity level is 375,180,000. The number of stereo matching operations of the proposed method is 59,116,200. This means that the computational load for stereo matching is decreased to 15.8%.

## 4. Conclusions

This paper presented a hierarchical stereo matching method to combine disparity estimation results in two-scale space efficiently. In general, the more pixels in a stereo view are examined, the more ambiguous correspondences are obtained. Furthermore, fine-detailed scene components may not be found accurately in a coarse scale. For these reasons, in our method we combine the disparity estimation results of high-frequency regions in the full-resolution image with the up-sampled disparity estimation results in the reduced-resolution image. More specifically, the initial matching cost of every pixel in the coarse scale is computed by using cross-based cost aggregation. High-frequency regions are extracted by DoG or edge components by a Canny operator in the full-resolution image. In the full-resolution image, we compute the initial matching cost of only the pixel in the high-frequency regions or edge components. Then the matching costs of the two-scale images are combined in fine scale. By applying EDP and refinement to the combined disparity results, we can obtain a precise final disparity map. According to the experimental results, the proposed algorithm provides a better performance in terms of both matching accuracy and computation efficiency than previous methods. 

Previous methods using the local plane sweeps often produce incorrect disparity results in areas with no textures and regions with repeated patterns. In addition, the method using disparity suggestion over scale space cannot obtain the accurate disparity result due to the incorrect initial disparity estimation in the lower resolution image. The method proposed can significantly reduce the matching ambiguity in a large untextured region because the disparity estimation is performed at a lower resolution. Also, the method proposed extracts disparity values from the high-frequency components, which are suitable for local stereo matching, in the high-resolution image. So incorrect disparity values are not propagated over the scale space. The method is proposed in only two scale spaces, which means a method for determining the optimal number of scale spaces according to the input image resolution is needed.

Occlusion is the key and challenging problem in stereo matching, because depth maps are significantly influenced by occlusion regions. In the near future, we will consider scene segmentation information such as boundaries and contours to solve the occlusion problem. In addition, more efficient optimization algorithms will be employed for computation performance improvement.

## Figures and Tables

**Table 1:** Quantitative evaluation results (error rate: %) for Middlebury database set.

Methods	Non-Occluded Pixels: Error > 1					Non-Occluded Pixels: Error > 2				
	Cones	Teddy	Baby3	Art	Lamp2	Cones	Teddy	Baby3	Art	Lamp2
Felzenszwalb [25]	15.2	18.7	13.0	23.3	32.0	7.8	11.4	7.0	16.5	26.0
Kolmogorov [26]	8.2	16.5	26.2	30.3	65.7	4.1	8.1	19.0	21.0	60.7
Cech [27]	7.2	15.8	17.4	18.8	36.7	4.4	10.2	9.7	11.2	27.1
Kostková [28]	7.2	13.5	14.2	17.9	31.5	5.3	10.1	8.2	13.0	26.7
Geiger [9]	5.0	11.5	10.8	13.3	17.5	2.7	7.3	4.5	8.7	10.4
Proposed method (Canny)	**4.7**	**8.5**	**4.9**	**10.8**	**7.9**	**1.6**	4.3	**2.1**	**8.0**	**4.0**
Proposed method (DOG)	4.8	8.7	5.2	**10.8**	8.0	1.7	**4.2**	2.3	8.1	4.1
